# The relationship between gut microbiota and susceptibility to type 2 diabetes mellitus in rats

**DOI:** 10.1186/s13020-023-00717-9

**Published:** 2023-05-05

**Authors:** Yongcheng An, Hongyu Dai, Yuhui Duan, Long Cheng, Lu Shi, Changhao He, Chen Wang, Yinglan Lv, Huimin Li, Huilin Zhang, Yan Huang, Wanxin Fu, Weiguang Sun, Baosheng Zhao

**Affiliations:** 1 Department of Pharmacology, School of Chinese Materia Medica, Beijing University of Chinese Medicine, Beijing, 102488 China; 2 Department of Pharmacognosy, School of Pharmacy, China Medical University, Shenyang, 110122 China; 3 School of Life Sciences, Beijing University of Chinese Medicine, Beijing, 102488 China; 4 Guangzhou Baiyunshan Xingqun Pharmaceutical Company Limited, Guangzhou, 510288 China; 5 Beijing Research Institute of Chinese Medicine, Beijing University of Chinese Medicine, Beijing, 100029 China

**Keywords:** Type 2 diabetes mellitus, Gut microbiota, 16S rRNA gene sequencing, Short-chain fatty acids, GPR41/GPR43

## Abstract

**Purpose:**

The purpose of this study is to investigate the relationship between the susceptibility to type 2 diabetes and gut microbiota in rats and to explore the potential mechanism involved.

**Methods:**

Thirty-two SPF-grade SD rats were raised as donor rats, and divided into control, type 2 diabetes mellitus (T2DM, fasting blood glucose ≥ 11.1 mmol/L), and Non-T2DM (fasting blood glucose < 11.1 mmol/L) groups. Feces were collected and prepared as fecal bacteria supernatants Diab (fecal bacteria supernatant of T2DM group rats), Non (fecal bacteria supernatant of Non-T2DM group rats), and Con (fecal bacteria supernatant of control group rats). Another seventy-nine SPF-grade SD rats were separated into normal saline (NS) and antibiotics (ABX) groups and given normal saline and antibiotics solutions, respectively. In addition, the ABX group rats were randomly separated into ABX-ord (fed with a 4-week ordinary diet), ABX-fat (fed with a 4-week high-fat diet and STZ *ip*), FMT-Diab (with transplanted fecal bacteria supernatant Diab and fed with a 4-week high-fat diet and STZ *ip*), FMT-Non (with transplanted fecal bacteria supernatant Non and fed with a 4-week high-fat diet and STZ *ip*), and FMT-Con (with transplanted fecal bacteria supernatant Con and fed with a 4-week high-fat diet and STZ *ip*) groups. Furthermore, the NS group was randomly divided into NS-ord (fed with a 4-week ordinary diet) and NS-fat (fed with a 4-week high-fat diet and STZ *ip*) groups. After this, the short-chain fatty acids (SCFAs) in the feces were detected using gas chromatography, and the gut microbiota were detected using 16S rRNA gene sequencing. Finally, G protein-coupled receptor 41 (GPR41) and GPR43 were detected by western blot and quantitative real-time polymerase chain reaction.

**Results:**

*G__Ruminococcus_gnavus_group* were more abundant in the FMT-Diab group compared to the ABX-fat and FMT-Non groups. The levels of blood glucose, serum insulin, total cholesterol, triglycerides, and low-density lipoprotein cholesterol were also higher in the FMT-Diab group compared to those of the ABX-fat group. Compared to the ABX-fat group, both the FMT-Diab and FMT-Non groups had higher contents of acetic and butyric acid, and the expression of GPR41/43 were significantly higher as well.

**Conclusions:**

*G__Ruminococcus_gnavus_group* might make rats more susceptible to T2DM; T2DM-susceptible flora transplantation increased the susceptibility to T2DM in rats. Additionally, gut microbiota-SCFAs-GPR41/43 may play a role in the development of T2DM. Lowering blood glucose by regulating gut microbiota may therefore become a new strategy for the treatment of T2DM in humans.

**Supplementary Information:**

The online version contains supplementary material available at 10.1186/s13020-023-00717-9.

## Background

In 2021, the 10th edition of the International Diabetes Federation's diabetes map showed that there were approximately 537 million diabetics worldwide, and with 140 million diabetics China has the most of any country. By 2045, the number of diabetes mellitus (DM) patients is expected to  increase to 783 million worldwide, more than 90% of which are type 2 diabetes mellitus (T2DM) [1]. T2DM is a chronic inflammatory multiple disease characterized by polydipsia, polyphagia, polyuria, and weight loss [2]. Long-term hyperglycemia caused by T2DM can lead to extensive vascular damage, affect the heart, eyes, kidneys and nerves, etc. and cause various complications, which seriously threaten human health and lives [3]. However, the pathogenesis of the disease remains unclear.

In recent years, researchers found that human pathological states are not solely regulated by genes but are the result of individuals’ relationships with their gut microbiota [4]. The number of gut microbiota in an average person is enormous, the species are numerous as well. Typically somewhere on the order of 10^14^ individual organisms inhabit the gut, comprising approximately 500 to 1000 different species [5, 6]. These gut microbiota are generally divided into beneficial, conditional pathogenic, and pathogenic bacteria according to their effects on the human body. These microbes compete with and restrict each other in the human intestine and jointly maintain intestinal homeostasis [7].

T2DM is classified as “*xiaoke*” in traditional Chinese medicine (TCM), which is divided into three types: “*shangxiao*”, “*zhongxiao*” and “*xiaxiao*”. “*Zhongxiao*” is mainly the lesion in the function of the digestive system. As an important part of the digestive system, gut microbiota are closely related to the development of T2DM [8], and other diseases such as obesity [9] are pathologically generated and developed in close relation to the gut microbiota as well. T2DM animal models are typically prepared using high-fat diet (HFD) and streptozotocin (STZ) intraperitoneal injection, however, this approach does not always lead to T2DM, and a similar phenomenon has been observed in clinical studies of humans. Not all people who eat high-calorie diets suffer from diabetes, and this fact may be related to the differences in individual gut microbiota composition. Some gut microbiota may predispose the host to T2DM and others may not [10, 11]. Certain types of gut microbiota produce short-chain fatty acids (SCFAs) in the intestine that can activate G protein-coupled receptor 41 (GPR41) and GPR43, thereby increasing the secretion of gastrointestinal hormones, reducing insulin resistance (IR), and lowering blood glucose levels [7]. Along this line, we found that the T2DM-susceptible flora transplantation made rats more susceptible to T2DM, and we further elucidated the pathological causality relationship between the gut microbiota, SCFAs, GPR41/GPR43 and T2DM.

## Materials and methods

### Animals

Male Sprague–Dawley rats (body weight, 200 ± 20 g) were purchased from SPF (Beijing) Biotechnology Co. Ltd (License No. SCXK (Jing) 2019–0010). The rats were housed under specific pathogen-free conditions at 20–24 ℃ with 40–70% humidity and a 12 h light/dark cycle in the Animal Experiment Center of Beijing University of Chinese Medicine. Donors rats, whose fecal material was used in transplantation experiments, were randomly divided into two groups: the control group (fed with an ordinary diet), and the HFD group (fed with a high-fat diet). Rats were fasted for 12 h after a 4-week course of their group’s diet after which time the HFD rats received 1% STZ citrate buffer solution (0.1 mmol/L, pH = 4.2 to 4.5) (Sigma Aldrich, USA) at a dose of 35 mg/kg [12], and the control rats received equal amounts of citrate buffer solution intraperitoneally. After three days, a cutoff value of 11.1 mmol/L was used to divide the HFD rats into a T2DM and a Non-T2DM group. T2DM rats’ feces were then collected as susceptible fecal bacteria Diab, Non-T2DM rats’ feces were collected as non-susceptible fecal bacteria Non and control rats' feces were collected as normal fecal bacteria Con.

### Preparation of pseudo-germ-free rats

**Fig. 1:**
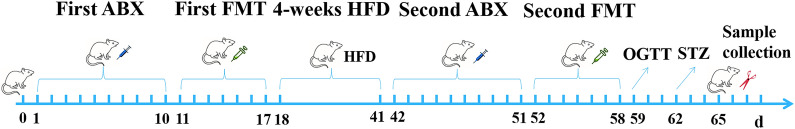
The experimental process

The experimental process is shown in Fig. 1. Seventy-nine SPF-grade SD rats were adaptively fed for 1 week, of which fifty-eight rats were given antibiotic solution [50 mg/kg Vancomycin, 100 mg/kg neomycin sulfate, 100 mg/kg metronidazole, 100 mg/kg ampicillin (Macklin)] by gavage, once a day, at a dosage of 1 mL/100 g of body weight, for 10 consecutive days (ABX group) [13, 14]. The other twenty-one rats were intragastrically administered with normal saline once a day for 10 days (NS group). Ten days later, the feces of both groups were collected for 16S rRNA gene sequencing: 1% solution of DEPC was sprayed in the air to eliminate RNase, and the rats’ naturally excreted feces were rapidly placed in RNase-free cryotubes and stored at − 80 °C [15].

### The first fecal microbiota transplant

The rats in the ABX group were randomly separated into 5 groups based on body weight: the ABX-ord group (who were fed an ordinary diet) (*n* = 10), the ABX-fat group (who were fed a high-fat diet) (*n* = 12), the fecal microbiota Diab transplantation (FMT-Diab) group (*n* = 12), the fecal microbiota Non transplantation (FMT-Non) group (*n* = 12) and the fecal microbiota Con transplantation (FMT-Con) group (*n* = 12). Fresh fecal samples from donor rats were collected, and each gram feces was resuspended in 5 mL of deionized water, stirred vortexed vigorously for 10 s, and then centrifuged at 3,500 rpm for 3 min at 4 °C. Any precipitate that could not pass through the gavage needle was discarded, and the resulting supernatant was used for FMT [16]. The supernatants of fecal bacteria Diab, Non and Con were prepared in the same way. The rats of FMT-Diab, FMT-Non and FMT-Con groups were intragastrically administered with their corresponding fecal bacteria supernatants (1 mL/100 g), once a day, for 7 consecutive days. Additionally, the rats of the ABX-ord, ABX-fat, and NS groups were intragastrically administered the same amount of deionized water 7 consecutive days, once a day. After this, the rats in the NS group were randomly divided into two groups based on body weight: the NS-ord group (fed an ordinary diet) (*n* = 10) and the NS-fat group (fed a high-fat diet) (*n* = 11). The feces of rats in every group were then collected for 16S rRNA testing.

### High-fat feeding

After FMT, the rats of the NS-fat, ABX-fat, FMT-Diab, FMT-Non, and FMT-Con groups were fed with a HFD for 4 weeks, and the NS-ord and ABX-ord groups were fed with an ordinary diet for 4 weeks. After 2 weeks and 4 weeks of feeding, feces were collected from above the groups for 16S rRNA determination as well.

### The second fecal microbiota transplant

After 4 weeks of HFD, the results of 16S rRNA showed that the gut microbiota of the rats had recovered, so a second FMT was performed. The FMT-Diab, FMT-Non, FMT-Con, ABX-ord and ABX-fat groups were intragastrically administered with antibiotic solution again for 10 days, once a day, and the rats in the NS-ord and NS-fat groups were intragastrically administered with normal saline. During this time, the blood glucose of the donor rats was also measured and is shown in Additional file 1: Table S1. Next, the rats of the FMT-Diab, FMT-Non, and FMT-Con groups were intragastrically administered with their corresponding fecal bacteria supernatant, and the rats of the ABX-ord, ABX-fat, NS-ord and NS-fat groups were intragastrically administered with the same amount of deionized water once a day for 7 days. After that, the feces of the rats in every group were collected for 16S rRNA determination.

### Oral glucose tolerance test and STZ injection

All rats were fasted and kept free of water overnight (12 h) before receiving oral glucose loading (2.0 g/kg). A glucose meter was used to measure blood glucose before and after loading with oral glucose for 15, 30, 60 and 120 min. The area under the curve (AUC) was calculated as follows:

$${\text{AUC }}\left( {{\text{mmol}}/{\text{h}}/{\text{L}}} \right) \, = { 1}/{2 } \times \, \left[ {\left( {{\text{BG}}0 \, + {\text{ BG15}}} \right) \, \times { 1}/{4 } + \, \left( {{\text{BG15 }} + {\text{ BG3}}0} \right) \, \times { 1}/{4 } + \, \left( {{\text{BG3}}0 \, + {\text{ BG6}}0} \right) \, \times { 1}/{2 } + \, \left( {{\text{BG6}}0 \, + {\text{ BG12}}0} \right)} \right]$$

BG: blood glucose value at different times.

Three days after oral glucose tolerance test (OGTT), the rats in the ABX-fat, NS-fat, FMT-Diab, FMT-Non and FMT-Con groups were intraperitoneally injected with 35 mg/kg STZ, and the same amount of citrate buffer solution was administered intraperitoneally to both the NS-ord and ABX-ord groups. Three days later, the modeling rate of rats was calculated and the feces of the rats in every group were collected for 16S rRNA testing as before. To make the experiment more meaningful, all rats with blood glucose ≥ 11.1 mmol/L were selected for mechanism validation.

### Biochemical and ELISA assay

Serum levels of total cholesterol (TC), triglycerides (TG), low-density lipoprotein cholesterol (LDL-C), and high-density lipoprotein cholesterol (HDL-C) were detected using various biochemical kits (Nanjing Jiancheng), and serum insulin levels were detected using an ELISA kit (Kete). Additionally, the homeostatic model assessment for insulin resistance (HOMA-IR) index was calculated as follows:

$${\text{HOMA}} - {\text{IR }} = {\text{ fasting insulin }}\left( {{\text{mU}}/{\text{L}}} \right) \, \times {\text{ FBG }}\left( {{\text{mM}}} \right)/{22}.{5}$$

### Histopathological examination

The rats’ pancreas tissues were fixed in 4% paraformaldehyde solution for 48 h, dehydrated with ethanol, embedded in paraffin, sectioned and stained with hematoxylin–eosin (H&E) to observe the pathological morphology under a microscope.

### 16S rRNA gene sequencing of gut microbiota

The 16S rRNA gene was sequenced based on an established method [17]. Briefly, DNA extraction and qRT-PCR amplification were performed first. And then, DNA was purified and sequenced. Finally, sequence processing was performed with an Illumina MiSeq PE300 platform [15].

### GC analysis

The standards for acetic acid (23.9 mg/mL), propionic acid (30.7 mg/mL), isobutyric acid (8.8 mg/mL), butyric acid (36.3 mg/mL) and 2-ethylbutyric acid (5.8 mg/mL) were prepared. In a 5 mL centrifuge tube, 200 mg of feces was placed, internal standard and appropriate amounts of ether were put, and hydrochloric acid was used to adjust the pH value. GC analysis was performed after vortexing and centrifuging samples, and passing them through a microporous membrane of 0.22 μm [18]. The chromatographic conditions were based on the established method (DB-FFAP capillary column (30 m × 0.25 mm × 0.25 μm; Agilent Technologies, Santa Clara, CA); carrier gas: high-purity nitrogen (purity > 99.99%); carrier gas flow rate: 1.0 mL/min; injection mode: split injection (split ratio 30:1); injection volume: 1 μL) [15].

### Quantitative real-time PCR

Intestine total RNA extraction was performed by TRIzol^®^ Reagent (Ambion), and reverse transcription with a Revert Aid First Stand cDNA Synthesis SuperMix (Novoprotein). Real-time quantitative PCR system with the SYBR qPCR SuperMix (Novoprotein) was used to detect gene expression. The PCR was performed in triplicate for each sample, and the data were expressed in arbitrary units after the normalization of β-actin expression levels. Additional file 1: Table S2 provided the primer sequences.

### Western blot analysis

The intestinal tissue protein concentration was determined by the bicinchoninic acid protein detection kit following weighing, grinding in liquid nitrogen, and lysing in RIPA Lysis Buffer (Beyotime). PVDF membrane was incubated in an enclosing solution for 2 h, and then incubated overnight at 4 °C in the required primary antibodies, including β-actin (1:20,000, 66,009-1-Ig, Proteintech), GPR41 (1:2000, OM184469, OmnimAbs, Alhambra), and GPR43 (1:1000, 19,952-1-AP, Proteintech). Following a wash with Tris-buffered saline as well as Tween 20, the membranes were incubated with the corresponding secondary antibodies and then exposed to chemiluminescence reagents and detection with an Amersham-enhanced chemiluminescence system. Image-Pro-Plus 6.0 was performed to quantify Western blot bands.

### Statistical analysis

All data were expressed as mean ± standard deviation (SD) values for each group and statistically analyzed using SPSS 22.0 and GraphPad Prism 8 software. For normally distributed data with homogeneous variances, the Wilcoxon rank sum test or one-way ANOVA (LSD *t*-test) was used, and for nonnormally distributed data, non-parametric testing was used. *P* < 0.05 was considered to indicate statistically significant test results for all tests.

## Results

### Summary of sequencing results

High-quality sequences ranging from 735,027 to 3,159,798 were retrieved from intestinal stool samples of all the rats and had an average length of 413 to 422 bp. In the alpha diversity of this study, rarefaction curves reached a stable point and Coverage surpassed 99.4%, indicating that the microbial community was near saturation, and most species could be detected in the sequencing amount. Alpha diversity measures include the rarefaction curve, Coverage, Shannon and Chao indexes, and those for β-diversity include the hierarchical clustering tree and PCoA.

### Successful establishment of pseudo-germ-free rats

**Fig. 2:**
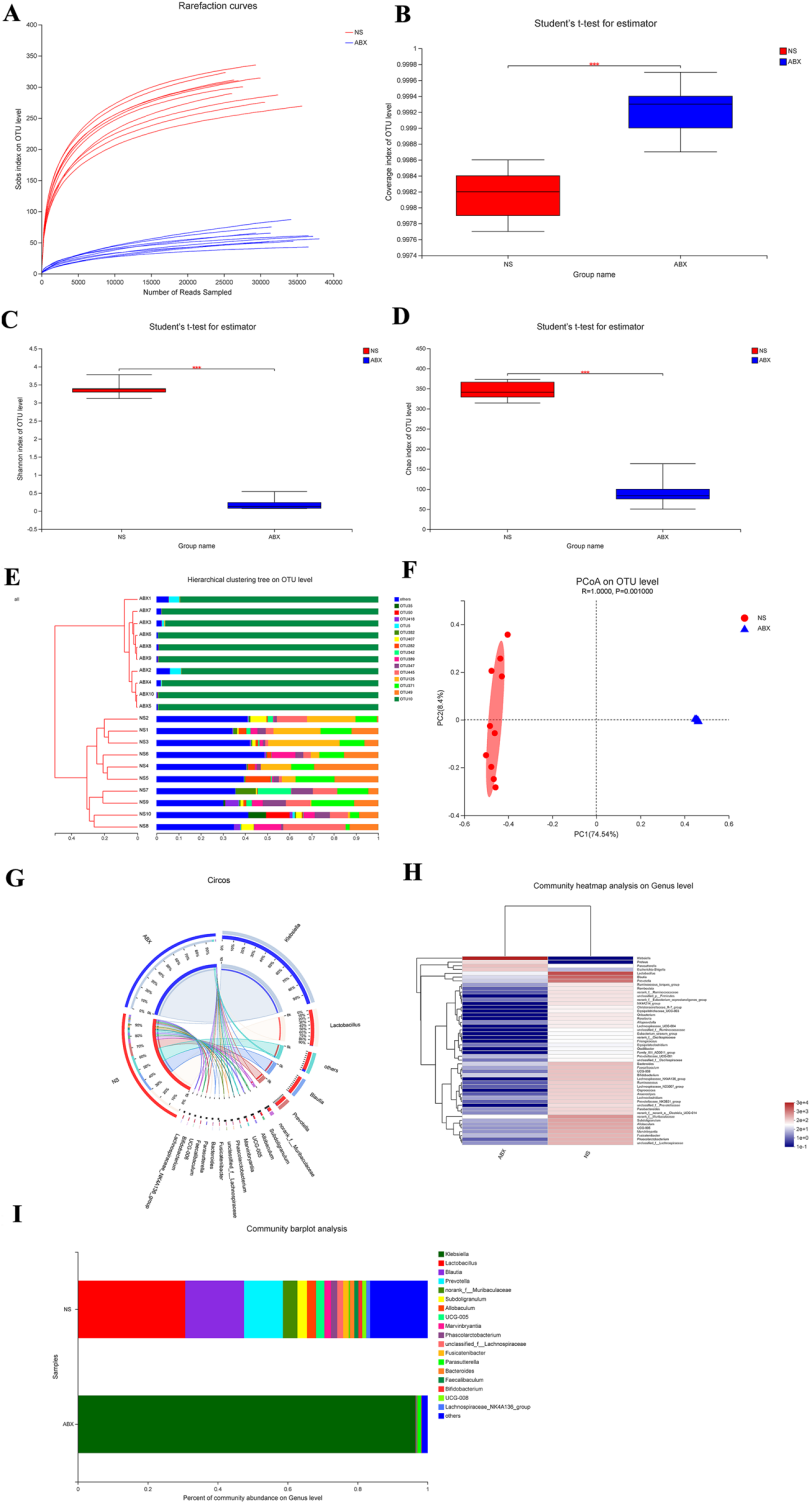
Diversity of fecal microbiota in the NS and ABX groups. **A** Rarefaction curve. **B–D** Bacteria that were different between the NS and ABX groups in the **B** Coverage, **C** Shannon, and **D** Chao indexes. Differences were assessed by the Wilcoxon rank-sum test. ^*^P < 0.05, ^**^P < 0.01, in comparison to the NS group. **E** Hierarchical clustering tree at the operational taxonomic unit (OTU) level. **F** Principal co-ordinate analysis (PCoA) at the OTU level. **G** Circos sample–species relation map. **H** Community heatmap analysis at the genus level. **I** Community barplot analysis

Alpha diversity measurement (Fig. 2A–D) showed that the abundance and diversity of gut microbiota in the ABX group were considerably lower than those in the NS group (*P* < 0.01), and the β-diversity results (Fig. 2E–F) demonstrated distinct clustering for each group at the OTU level. Circos, heatmap and barplot analysis (Fig. 2G–I) all indicated that the bacterial genera of the ABX group was significantly less diverse than that of the NS group as well. In conclusion, the biological abundance and biodiversity of gut microbiota in the ABX group were substantially reduced, and the pseudo-germ-free rats were successfully established.

We used Wilcoxon rank-sum tests to investigate the differences between the above two groups' fecal bacterial communities (selecting species with the top 15 mean sums, *P* < 0.05, Additional file 2: Fig. S1 A-E), and compared to the NS group, *p__Proteobacteria*, *c__Gammaproteobacteria*, *o__Enterobacterales*, *f__Enterobacteriaceae* and *f__Morganellaceae* were evidently more numerous in the ABX group. At the genus level, *g__Klebsiella* was also evidently more abundant, but the other species of this genus were evidently less. So we then determined the altered specific bacterial taxa between the two groups (Additional file 2: Fig. S1F) by utilizing a linear discriminant analysis (LDA) effect size (LEfSe) algorithm (LDA values of > 2 with *P* < 0.05). The NS group had 16 species with proportions exceeding 1%, which are listed in Additional file 1: Table S3, but the ABX group only had 2.

### Dynamic changes in gut microbiota after the first FMT

**Fig. 3:**
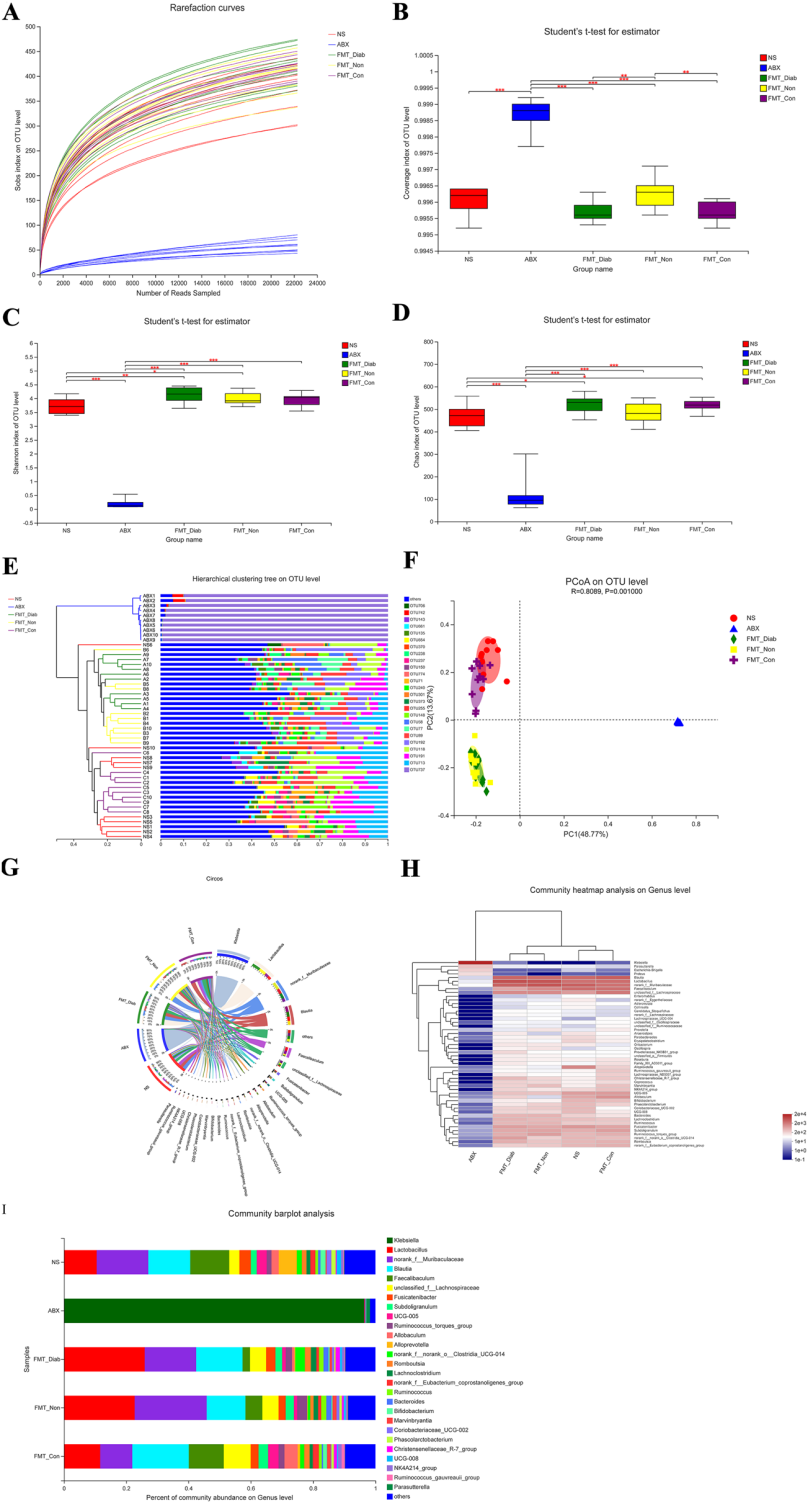
Diversity of fecal microbiota in the NS, ABX, FMT-Diab, FMT-Non and FMT-Con groups. **A** Rarefaction curve. **B–D** Bacteria that were different among the groups in the **B** Coverage, **C** Shannon index and **D** Chao index. Differences were assessed by the Wilcoxon rank-sum test. ^*^P < 0.05, ^**^P < 0.01. **E** Hierarchical clustering tree at the operational taxonomic unit (OTU) level. **F** Principal co-ordinate analysis (PCoA) at the OTU level. **G** Circos sample–species relation map. **H** Community heatmap analysis at the genus level. **I** Community barplot analysis

Alpha diversity measurements (Fig. 3A–D) also showed that the abundance and diversity of the gut microbiota in the ABX group were evidently lower than in the NS group (*P* < 0.01). The richness and diversity of species in the FMT-Diab, FMT-Non, and FMT-Con groups were significantly higher than the ABX group (*P* < 0.01), which demonstrated that the abundance and diversity of gut microbiota in the pseudo-germ-free rats were distinctly enhanced after FMT. The β-diversity results showed that the groups were strongly clustered, and that the NS and FMT-Con groups had a certain similarity at the OTU level. Furthermore, circos, heatmap and barplot (Fig. 3E–I) showed that the groups were distinctly different from each other at the genus level. The NS and FMT-Con groups had some similarity. Based on the above analysis, fecal microbiota did successfully colonize the intestines of the pseudo-germ-free rats after FMT.

### Dynamic changes in gut microbiota structure after 2 weeks of HFD

Alpha diversity measurements (Additional file 3: Fig. S2A-D) indicated that the richness of the gut microbiota in the ABX-ord group was obviously lower than that in the NS-ord group (*P* < 0.01), but the diversity of the gut microbiota showed no remarkable difference, implying that with the extension of feeding time, the gut microbiota in the pseudo-germ-free rats became progressively revived. The abundance and diversity of the gut microbiota in the ABX-fat group were both evidently lower than in the ABX-ord group (*P* < 0.01), which suggests that HFD was not conducive to the self-recovery of the gut microbiota. At the OTU and genus level, the results showed that the NS-fat, ABX-fat, FMT-Diab, FMT-Non, and FMT-Con groups had close similarity, and each group had certain clustering that was readily observed (Additional file 3: Fig. S2 E-I). In addition, the differences in the gut microbiota among the groups were lower than those after the first FMT.

### Dynamic changes in gut microbiota structure after 4 weeks of HFD

The 16S rRNA sequencing results showed that the clustering of the groups was poor, and the differences in the gut microbiota among groups became smaller than after two weeks of HFD (Additional file 4: Fig. S3 A-I). Since the gut microbiota of the rats had self-recovery ability, the difference among the groups steadily diminished with time, and we therefore executed a second FMT.

### Dynamic changes in gut microbiota after the second FMT

The 16S rRNA results after the second FMT (Additional file 5: Fig. S4 A-I) showed that the abundance and diversity of the gut microbiota communities in the NS-fat, FMT-Diab, FMT-Non, and FMT-Con groups were signally higher compared to the ABX-fat group (*P* < 0.01). The ABX-ord group was similar in depth to the ABX-fat group, with *g__Klebsiella* being the most abundant bacterial genus. In conclusion, the second FMT effectively improved the composition of the gut microbiota of rats in every group.

### The effects of FMT on glucose and lipid metabolism and IR

**Fig. 4:**
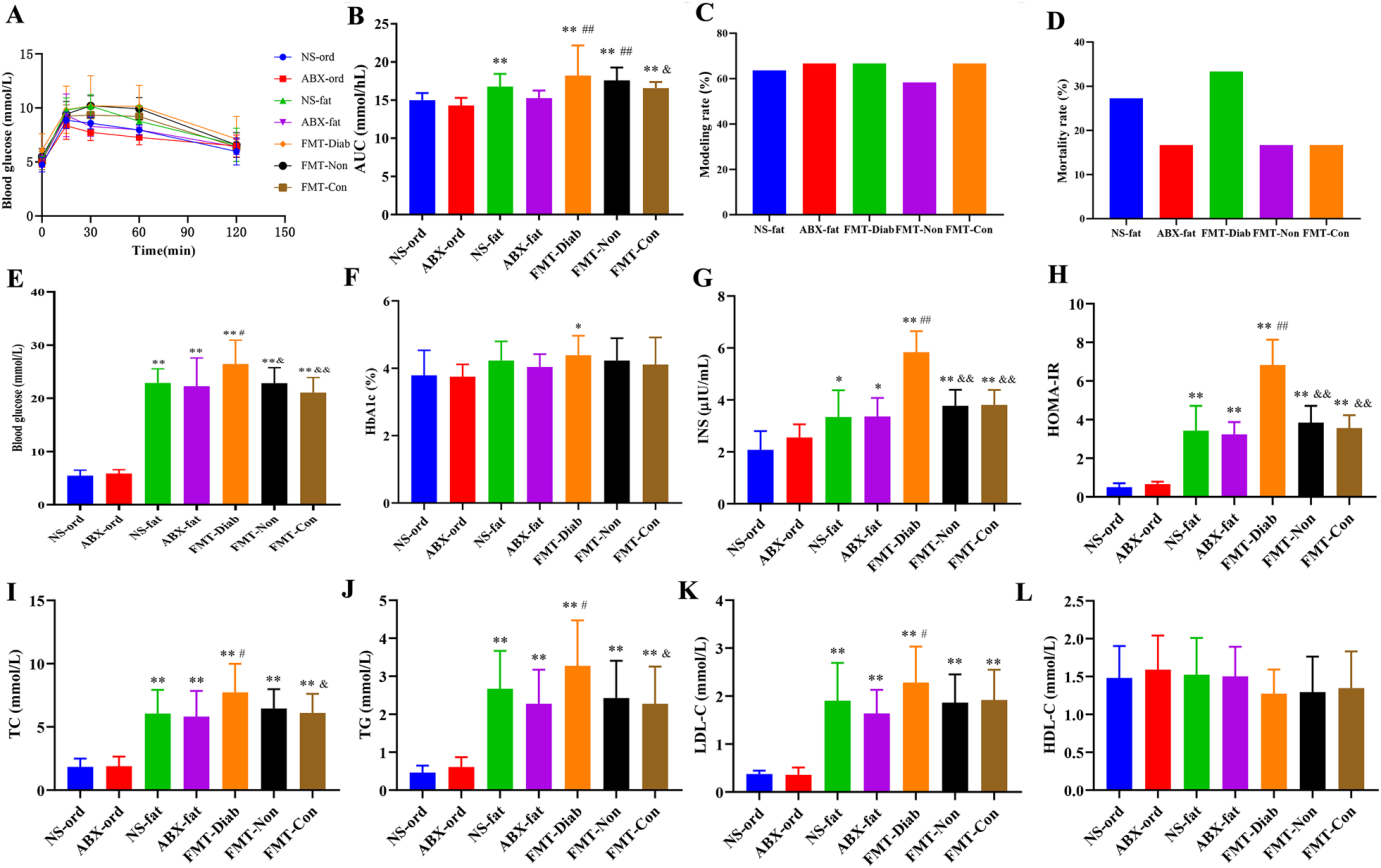
The effects of HFD and STZ on glycolipid metabolism. **A** Blood glucose levels were measured before 0 min and at 15, 30, 60, 90, and 120 min after glucose loading. **B** AUC of the OGTT. **C** molding rate. **D** mortality rate. **E** FBG. **F** HbA1c. **G** insulin. **H** HOMA-IR. **I** TC. **J** TG. **K** LDL-C. **L** HDL-C. Data are shown as mean ± SD.^*^P < 0.05, ^**^P < 0.01 vs. ABX-ord group; ^#^*P* < 0.05, ^##^*P* < 0.01 vs. ABX-fat group; ^&^*P* < 0.05, ^&&^*P* < 0.01 vs. FMT-Diab group

OGTT assay was used to evaluate the rats’ glucose tolerance (Fig. 4A–B). The NS-fat, FMT-Diab, FMT-Non, and FMT-Con groups showed significantly elevated glucose excursions following glucose challenge compared to the ABX-ord group (*P* < 0.01), and the FMT-Diab group increased its glucose excursions more than the ABX-fat group (*P* < 0.01). Compared to the ABX-fat group, the molding rate (fasting blood glucose ≥ 11.1 mmol/L) of the FMT-Non group was lower, and the mortality rate of the FMT-Diab was higher. For blood markers, the levels of blood glucose, serum insulin, HOMA-IR, TC, TG, and LDL-C in the FMT-Diab group were clearly higher than those in the ABX-fat group (*P* < 0.05), and the level of HbA1c showed an upward trend. However, the level of HbA1c in the FMT-Diab group was distinctly higher than in the ABX-ord group (*P* < 0.05). These results indicate that T2DM-susceptible flora transplantation could increase the level of blood glucose, decrease the level of serum insulin, and promote IR, slowing down lipid metabolism in rats.

### The effect of FMT on pancreatic islet histopathological alterations

**Fig. 5:**
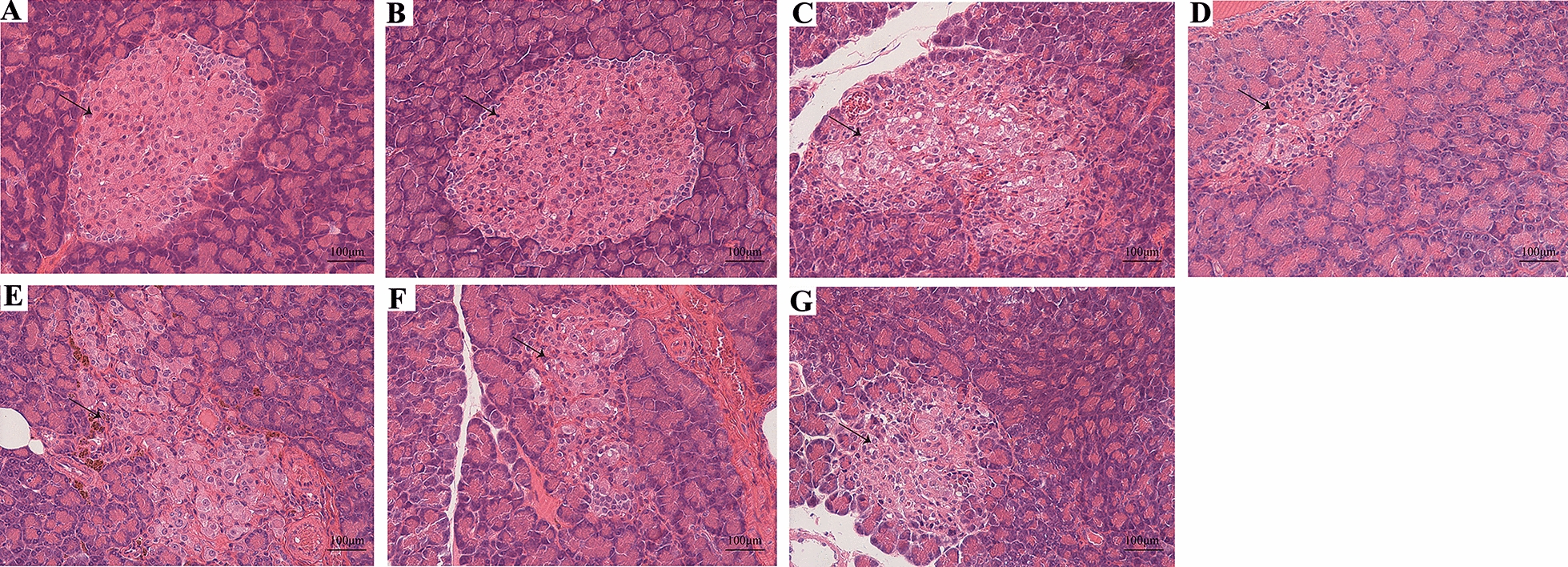
Micrographs of rat pancreas specimens by H&E staining in the **A** NS-ord, **B** ABX-ord,** C** NS-fat, **D** ABX-fat, **E** FMT-Diab, **F** FMT-Non, and **G** FMT-Con (Magnification: × 200). Scale bar: 100 μm

As shown in Fig. 5, the rats’ pancreatic islets were plump and elliptical, and the exocrine acinar cells were around the islets, which themselves showed no abnormal pathological changes in the NS-ord and ABX-ord groups. A diminished number and volume of islets, islet cell necrosis, vacuolar degeneration, and fibrous tissue hyperplasia were observed in the entire field of vision in the NS-fat group. Furthermore, the volume of islets in the ABX-fat group was markedly reduced, and their structure was disordered and accompanied by fibrous tissue hyperplasia. In the FMT-Diab group, the islet structure was also disordered, and the hemosiderin was deposited, accompanied by fibrous tissue hyperplasia and severe inflammation. For the FMT-Non group, there was likewise a decreased number of islets, reduced volume, disordered structure, and hyperplasia of fibrous tissue. Finally, in the FMT-Con group, the number of islets  was decreased, the volume was reduced, the structure was disordered, and a small amount of vacuolar degeneration occurred.

### The effects of FMT on the composition of the colonic microbiota

**Fig. 6:**
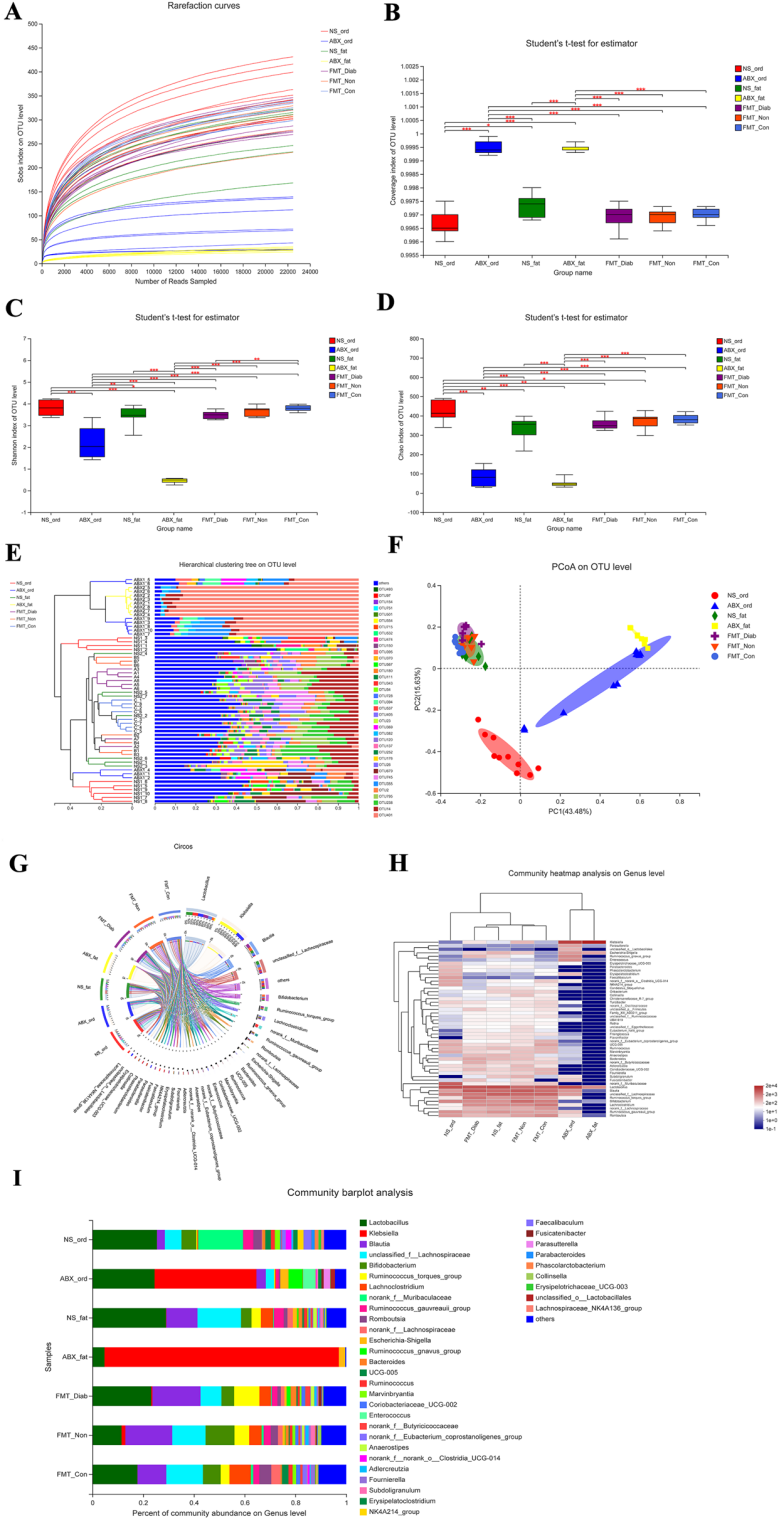
Diversity of fecal microbiota in the NS-ord, ABX-ord, NS-fat, ABX-fat, FMT-Diab, FMT-Non and FMT-Con groups. **A** Rarefaction curve. **B–D** Bacteria that were different among the groups in the **B** Coverage, **C** Shannon index and **D** Chao index. Differences were assessed by the Wilcoxon rank-sum test. ^*^P < 0.05, ^**^P < 0.01. E Hierarchical clustering tree at the operational taxonomic unit (OTU) level. **F** Principal co-ordinate analysis (PCoA) at the OTU level. **G** Circos sample—species relation map. **H** Community heatmap analysis at the genus level. **I** Community barplot analysis

Alpha diversity measurements (Fig. 6A–D) showed that the richness of gut microbiota was not substantially different between the ABX-ord and ABX-fat groups but that the diversity in the ABX-fat group was distinctly lower than in the ABX-ord group (*P* < 0.01). The richness and diversity of communities in the FMT-Diab, FMT-Non, and FMT-Con groups were prominently higher compared to the ABX-fat group as well (*P* < 0.01). Neither the diversity nor richness of the FMT-Con group and NS-fat group were clearly different. Similarly, the abundance and diversity of communities in the FMT-Diab group were not evidently different compared to the FMT-Non group.

The β-diversity results (Fig. 6E–F) showed that the NS-ord group had distinct clustering and was obviously different from other groups at the OTU level. At the genus level, the ABX-ord group was similar to the ABX-fat group, and the NS-fat, FMT-Diab, FMT-Non, and FMT-Con groups were also all similar to one another (Fig. 3G–I), because they were obviously affected by the STZ, which exceeded the effect of FMT and resulted in a convergence of the flora in each group.

**Fig. 7:**
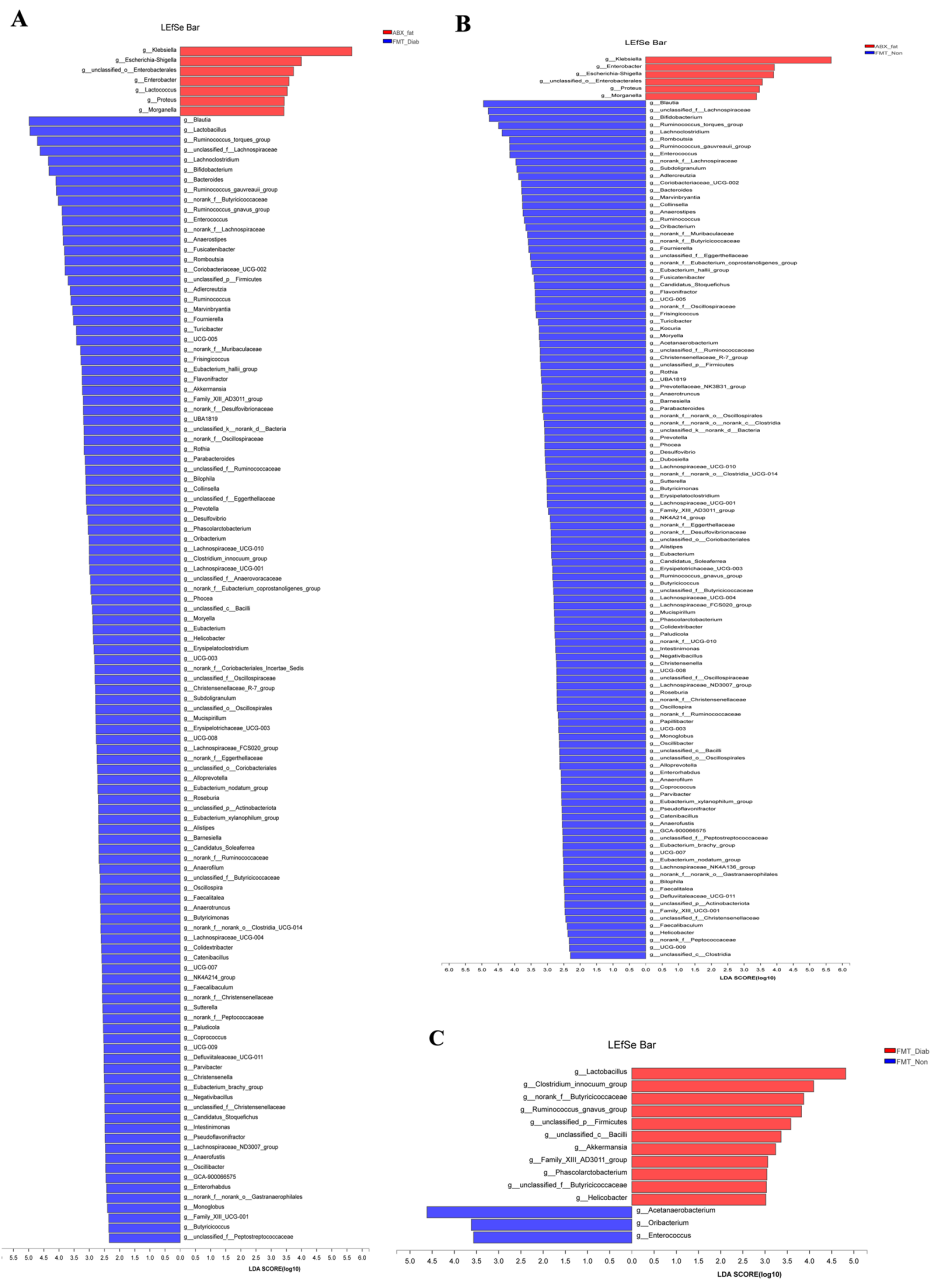
LDA scores of gut microbiota for the **A** ABX-fat and FMT-Diab groups; **B** ABX-fat and FMT-Diab groups; **C** FMT-Diab and FMT-Non groups at the genus level

To understand the differences in every group further and to obtain the bacterial genera that may be linked to T2DM development and occurrence, LEfSe multi-level discriminant analysis was conducted. The genera proportions exceeding 1% can be found in Additional file 1: Table S4. In Fig. 7, compared to the ABX-fat group, *g__Klebsiella* and *g__Escherichia-Shigella* were lower in the FMT-Diab and FMT-Non group, and *g__Blautia*, *g__Lactobacillus*, *g__Ruminococcus_torques_group*, *g__unclassified_f__Lachnospiraceae*, *g__Lachnoclostridium*, *g__Bifidobacterium*, *g__Ruminococcus_gauvreauii_group*, *g__Bacteroides*, *g__Fusicatenibacter*, *g__Ruminococcus_gnavus_group*, *g__Coriobacteriaceae_UCG-002*, *g__norank_f__Butyricicoccaceae*, *g__norank_f__Lachnospiraceae*, *g__Anaerostipes* and *g__Romboutsia* were higher in the FMT-Diab group. Among them, *g__Klebsiella* reached 92.32% in the ABX-fat group, and *g__Lactobacillus* reached 23.23%, and *g__Blautia* reached 18.93% in the FMT-Diab group. In addition, compared to the FMT-Diab group, the *g__Lactobacillu*s, *g__norank_f__Butyricicoccaceae* and *g__Ruminococcus_gnavus_group* were noticeably lower in the FMT-Non group. Our team's previous study showed that *g__Ruminococcus_gnavus_group* was markedly different in the donor T2DM and Non-T2DM rats [15], and it was still markedly different after FMT. Therefore, we consider *g__Ruminococcus_gnavus_group* to be a specific genus that affects T2DM outcomes.

### Mechanism validation

**Fig. 8:**
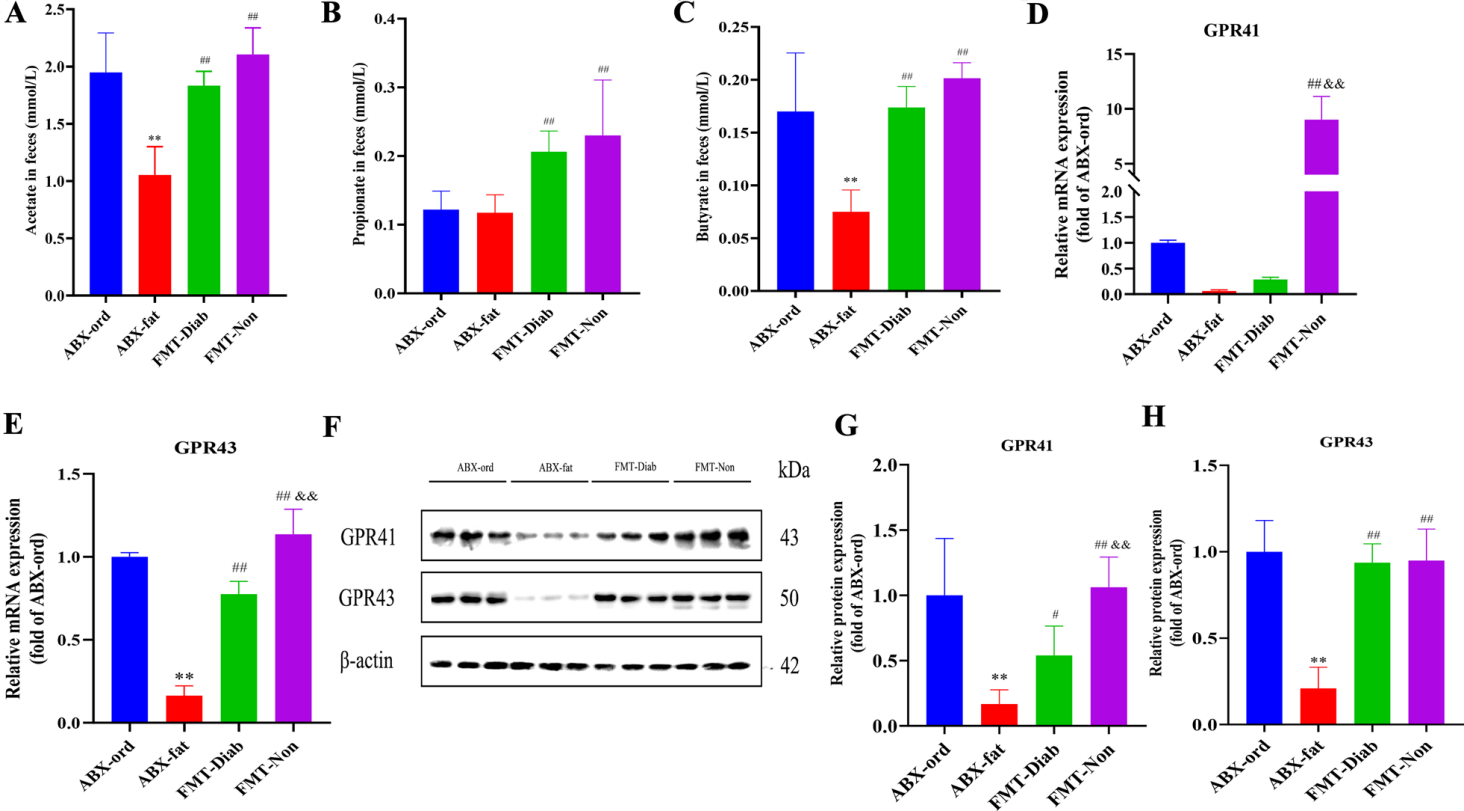
The mechanism was investigated using gas chromatography, Western blot, and qRT-PCR. **A–D** SCFA content in the ABX-ord, ABX-fat, FMT-Diab, and FMT-Non groups. **A** Acetate. **B** Propionate. **C** Butyrate. **D–E** GPR41 and GPR43 mRNA expression (*n* = 3). **F–H** GPR41 and GPR43 protein expression. Data were shown as mean ± SD. ^*^P < 0.05, ^**^P < 0.01 vs. ABX-ord group; ^#^*P* < 0.05, ^##^*P* < 0.01 vs. ABX-fat group; ^&^*P* < 0.05, ^&&^*P* < 0.01 vs. FMT-Diab group

Researchers have found that T2DM is associated with SCFAs, primarily acetic acid, propionic acid, and butyric acid, which play a crucial role in regulating glycolipid metabolic disorders, improving IR, and treating obesity and other metabolic diseases. In Fig. 8A–C, we can see that compared with the ABX-ord group, the contents of acetic and butyric acid were distinctly lower in the ABX-fat group (*P* < 0.01). The contents of acetic, propionic and butyric acid in the FMT-Diab and FMT-Non groups were significantly increased compared to the ABX-fat group as well (*P* < 0.01). In addition, compared to the FMT-Diab group, the FMT-Non group had an increasing trend.

QRT-PCR and WB assay was performed to examine the connection between SCFAs and T2DM by detecting GPR41 and GPR43 mRNA and protein levels. In Fig. 8D–H, the results from the WB agreed with those obtained from qRT-PCR. Compared to the ABX-ord group, the expression of GPR41/43 in the ABX-fat group were remarkably lower. Compared to the ABX-fat group, the expression of GPR41/43 were higher in the FMT-Diab and FMT-Non groups, and compared to the FMT-Diab group, the expression of GPR41/43 in the FMT-Non group were higher. These results indicate that T2DM-susceptible flora transplantation can reduce the production of SCFAs and the expression of GPR41/43 in the intestines of rats.

## Discussion

Gut microbiota are closely related to many metabolic diseases, including T2DM and hepatic steatosis. In this study we found moderate intestinal microbial disorders in T2DM rats[8], accompanied by a significant decrease in total and probiotic bacteria; and an increase in harmful bacteria in the intestine [19], consistent with previous studies. In TCM, fecal medicines such as Wulingzhi are widely used in clinical practice and have good therapeutic effects [20]. In addition, other related studies have found that FMT can alleviate ulcerative colitis [21], obesity and metabolic syndrome [22]. According to clinical observations, the susceptibility of each individual to T2DM is related to his or her innate endowment, which may be related to the composition of his or her gut microbiota. These studies have shown that gut microbiota play a vital role in the onset and development of T2DM and that changing the gut microbiota may help to control the T2DM process [11, 23].

In the present study, the pseudo-germ-free rat model was prepared by gavage of antibiotic solution, and our 16S rRNA  sequencing results showed that the species and richness of the gut microbiota were greatly reduced and that the abundance of *g__Klebsiella* was only distinctly increased in the ABX group. *G__Klebsiella*, is an opportunistic pathogen living in the upper respiratory and digestive tract of the body. When the body's immunity is reduced, *g__Klebsiella* can proliferate rapidly, impairing organs such as the lungs and causing infection [24]. After we performed the first FMT, the 16S rRNA analysis results revealed that fecal microbiota did successfully colonize the intestines of the pseudo-germ-free rats. However, along with a 4-week course of diet, the gut microbiota gradually began to recover as the pseudo-germ-free rats could not be kept in an absolutely sterile environment, combined with the rats themselves also have a certain resilience. We therefore performed a second antibiotic administration to remove the gut microbiota and a second FMT to colonize the flora in the intestines of these rats.

T2DM is a disease of faulty glucose and lipid metabolism disorder. In this study, our experiment data showed that transplanted fecal bacteria Diab increased blood glucose, serum insulin, HOMA-IR, TC, TG, and LDL-C of otherwise rats, indicating that fecal bacteria Diab made rats more susceptible to T2DM. Fecal bacteria Non did not alleviate or reduce the risk of T2DM in rats in this experiment. However, under the same modeling conditions, the blood glucose, modeling rate and serum insulin levels in the FMT-Non group were obviously lower than those in the FMT-Diab group, indicating that fecal bacteria Non made the rats less susceptible to T2DM to a certain extent. We speculate that this is to be due to the strong destructive effect of STZ on the pancreas, which is overwhelms the ability of FMT to regulate blood glucose, but another possible reason is that the FMT-Non group received Non-T2DM rats (4-week HFD, STZ *ip* and blood glucose < 11.1 mmol/L) fecal bacteria, whose blood glucose was still higher than that of normal rats, and this would also explain the similarity in the gut microbiota of the FMT-Diab and FMT-Non groups. In the future, we intend to separate and screen different gut microbiota explicitly so as to explore the role of different gut microbiota in the occurrence and development of T2DM futher.

Previous studies have found that the content of *g__Ruminococcus_gnavus_group* in T2DM rats is remarkably higher than in control and Non-T2DM groups [15]. This study further examined the association between fecal bacteria Diab and T2DM susceptibility through FMT, and documented the relationship between T2DM susceptibility and gut microbiota. Among them, *g__Ruminococcus_gnavus_group* was found to be specific for T2DM, and its abundance in T2DM rats and the FMT-Diab group was evidently higher than in other groups. *G__Ruminococcus_gnavus_group* is also positively correlated with fasting blood glucose [25], which can lead to obesity by causing cells to absorb too much sugar [26]. SCFAs are organic acids containing 2 to 5 carbon atoms and are mainly produced by bacterial fermentation of oligosaccharides, polysaccharides, peptides, proteins, and glycoproteins in the intestine. They are also considered to be important markers for the diagnosis of chronic metabolic diseases [27]. These compound are not only absorbed as nutrients but also influence various physiological effects of the intestine [28]. Our GC analysis showed that the contents of SCFAs in the ABX-fat group were significantly lower than those in the ABX-ord group, indicating that T2DM inhibited the production of SCFAs. The contents of SCFAs in the FMT-Diab and FMT-Non groups were significantly higher compared to the ABX-fat group as well, suggesting that FMT can increase the production of SCFAs. The contents of SCFAs in the FMT-Non group also showed an increasing trend compared to the FMT-Diab group, indicating that the role of fecal microbiota Non in promoting the production of SCFAs was limited, though further experiments are needed to verify.

GPR41/43 are specific receptors for SCFAs, which are associated with metabolic diseases such as T2DM and appetite dysregulation [29]. SCFAs activate GPR41, which promotes the secretion of insulin and glucagon-like peptide-1 (GLP-1), thereby regulating lipid and energy metabolism and reducing peripheral blood glucose levels [30, 31].  In addition, GPR43 has been found to be a sensor of excess dietary energy, thereby controlling the body's energy utilization and maintaining a stable metabolic environment. Furthermore, SCFA-mediated activation of GPR43 inhibits insulin signaling in adipocytes, thereby inhibiting fat accumulation in adipose tissue and facilitating the metabolism of unbound lipids and glucose in other tissues [32]. The expression of GPR41/43 were significantly increased in the colon tissue of both the FMT-Diab and FMT-Non groups in line with the trend in the SCFAs in the feces as compared to the ABX-fat group, which showed that FMT can significantly improve SCFA contents and GPR41/43 expression. Compared to the FMT-Diab group, the elevated levels of these substances in the FMT-Non group also indicated that T2DM susceptible flora transplantation could also reduce the content of SCFAs and the expression of GPR41/43 in rats.

## Conclusion

In conclusion, this study showed that the increase of T2DM-susceptibility bacteria such as *g__Ruminococcus_gnavus_group* and the decrease of beneficial bacteria such as *g__Prevotella* and *g__Allobaculum* lead to a decrease in SCFAs and GPR41/43 expression, thus inducing T2DM. We therefore conclude that "gut microbiota – SCFAs – GPR41/43" is involved in the pathological process of T2DM, and this finding may provide a new strategy for the treatment of diabetes and a new direction for the research and development of new hypoglycemic drugs.

## Supplementary Information

**Additional file 1: Table S1.** The blood glucose of the donor rats.** Table S2**. The primer sequences for qRT-PCR.** Table S3**. The proportion exceeded 1% of species between the NS and ABX groups.** Table S4**. The proportion of genera in each group exceeds 1%.

**Additional file2: Figure S1.** Gut microbiota composition profiles in the NS and ABX groups. **A–E** Bacteria were evidently different between the NS and ABX groups at the **A** phylum level, **B** class level, **C** order level, **D** family level, and **E** genus level. The data are presented as the relative abundance (%) of the first 15 taxa with the most significant differences between the two groups. Statistical analysis was performed by the Wilcoxon rank-sum test. ^*^P < 0.05, ^**^P < 0.01, and ^***^P < 0.001, in comparison with the NS group. **F** LDA scores of gut microbiota for the NS and ABX groups at the genus level.

**Additional file 3: Figure S2.** Diversity of fecal microbiota in the NS-ord, ABX-ord, NS-fat, ABX-fat, FMT-Diab, FMT-Non and FMT-Con groups. **A** Rarefaction curve. **B–D** Bacteria that were different between the NS and ABX groups in the **B** Coverage, **C** Shannon index and **D** Chao index. Differences were assessed by the Wilcoxon rank-sum test. ^*^P < 0.05, ^**^P < 0.01. **E** Hierarchical clustering tree at the operational taxonomic unit (OTU) level. **F** Principal co-ordinate analysis (PCoA) at the OTU level. **G** Circos sample–species relation map. **H** Community heatmap analysis at the genus level. **I** Community barplot analysis.

**Additional file 4: Figure S3.** Diversity of fecal microbiota in the NS-ord, ABX-ord, NS-fat, ABX-fat, FMT-Diab, FMT-Non and FMT-Con groups. **A** Rarefaction curve. **B–D** Bacteria that were different among the groups in the **B** Coverage, **C** Shannon index and **D** Chao index. Differences were assessed by the Wilcoxon rank-sum test. ^*^P < 0.05, ^**^P < 0.01. **E** Hierarchical clustering tree at the operational taxonomic unit (OTU) level. **F** Principal co-ordinate analysis (PCoA) at the OTU level. **G** Circos sample–species relation map. **H** Community heatmap analysis at the genus level. **I** Community barplot analysis.

**Additional file 5: Figure S4.** Diversity of fecal microbiota in the NS-ord, ABX-ord, NS-fat, ABX-fat, FMT-Diab, FMT-Non and FMT-Con groups. **A** Rarefaction curve. **B–D** Bacteria that were different among the groups in the **B** Coverage, **C** Shannon index and **D** Chao index. Differences were assessed by the Wilcoxon rank-sum test. ^*^P < 0.05, ^**^P < 0.01. **E** Hierarchical clustering tree at the operational taxonomic unit (OTU) level. **F** Principal co-ordinate analysis (PCoA) at the OTU level. **G** Circos sample–species relation map. **H** Community heatmap analysis at the genus level. **I** Community barplot analysis.

## Data Availability

All data used and analyzed in the current study are available from the corresponding author upon request.

## Abbreviations

T2DM: Type 2 diabetes mellitus
NS: Normal saline
GPR41: G protein-coupled receptor 41
GPR43: G protein-coupled receptor 43
SCFAs: Short-chain fatty acids
FBG: Fasting blood glucose
TC: Total cholesterol
TG: Triglyceride
LDL-C: Low-density lipoprotein cholesterol
DM: Diabetes mellitus
STZ: Streptozotocin
HFD: High-fat diet
GLP-1: Glucagon-like peptide-1
FMT: Fecal microbiota transplantation
