# Supplementary material for: The relationship between gut microbiota and susceptibility to type 2 diabetes mellitus in rats
Source: Chin Med. 2023 May 5;18:49. doi: 10.1186/s13020-023-00717-9 (PMC10161507; doi:10.1186/s13020-023-00717-9)
Supplement: Supplementary file 1 — Additional file 1: Table S1. The blood glucose of the donor rats. Table S2. The primer sequences for qRT-PCR. Table S3. The proportion exceeded 1% of species between the NS and ABX groups. Table S4. The proportion of genera in each group exceeds 1%. [file 13020_2023_717_MOESM5_ESM.docx]

**Additional files**

Table S1 The blood glucose of the donor rats.

| Group | *n* | Blood glucose（mmol/L） |
| --- | --- | --- |
| Con | 10 | 6.04±0.49 |
| T2DM | 10 | 20.87±2.98^**^ |
| Non-mod | 8 | 7.96±1.01^*##^ |

Table S2 The primer sequences for qRT-PCR

| Primer | Forward (5'-3') | Reverse (5'-3') |
| --- | --- | --- |
| *GPR41* | TCTGCTCCTCTTCCTGCCATTCC | CGTTCTATGCTCACCGTCATCAGG |
| *GPR43* | TGCACCATCGTCATCATCGTTCAG | ACCAGGCACAGCTCCAGTCG |
| *β-actin* | GCCCCTCTGAACCCTAAG | ATGTCACGCACGATTTCC |

Table S3 The proportion exceeded 1% of species between the NS and ABX groups.

| Species name | NS-Mean(%) | ABX-Mean(%) |
| --- | --- | --- |
| *g__Lactobacillus* | 31.93 | 0.1175 |
| *g__Blautia* | 16.35 | 0.06694 |
| *g__Prevotella* | 10.93 | 0.04336 |
| *g__norank_f__Muribaculaceae* | 4.165 | 0.1817 |
| *g__Allobaculum* | 2.576 | 0.01377 |
| *g__UCG-005* | 2.431 | 0.01283 |
| *g__Subdoligranulum* | 2.377 | 0.0205 |
| *g__Marvinbryantia* | 1.8 | 0.01242 |
| *g__unclassified_f__Lachnospiraceae* | 1.773 | 0.013 |
| *g__Phascolarctobacterium* | 1.77 | 0.004025 |
| *g__Fusicatenibacter* | 1.443 | 0.02909 |
| *g__Bacteroides* | 1.393 | 0.04946 |
| *g__Faecalibaculum* | 1.167 | 0.02043 |
| *g__Lachnospiraceae_NK4A136_group* | 1.162 | 0.0002933 |
| *g__Bifidobacterium* | 1.082 | 0.04726 |
| *g__UCG-008* | 1.077 | 0.005186 |
| *g__Klebsiella* | 0.0003298 | 96.48 |
| *g__Parasutterella* | 0.2221 | 1.112 |

Table S4 The proportion of genera in each group exceeds 1%.

| Species name | NS-ord  -Mean(%) | ABX-ord  -Mean(%) | NS-fat  -Mean(%) | ABX-fat  -Mean(%) | FMT_Diab  -Mean(%) | FMT_Non  -Mean(%) | FMT_Con  -Mean(%) |
| --- | --- | --- | --- | --- | --- | --- | --- |
| *g__Lactobacillus* | 25.49 | 24.51 | 29.05 | 4.824 | 23.23 | 11.52 | 17.74 |
| *g__norank_f__Muribaculaceae* | 17.68 | 0.0004446 | 0.3227 | 0.0005558 | 0.3646 | 0.8042 | 0.9916 |
| *g__unclassified_f__Lachnospiraceae* | 6.567 | 3.283 | 17.11 | 0.0005558 | 8.258 | 13.18 | 14.43 |
| *g__Bifidobacterium* | 5.809 | 0.003112 | 4.149 | 0.01556 | 5.001 | 11.37 | 7 |
| *g__Ruminococcus_gauvreauii_group* | 4.022 | 1.098 | 3.905 | 0.0005558 | 2.223 | 2.789 | 2.381 |
| *g__Romboutsia* | 3.347 | 0.5638 | 1.439 | 0.002223 | 1.357 | 2.815 | 4.72 |
| *g__Blautia* | 3.04 | 3.683 | 12.27 | 0.004446 | 18.93 | 18.43 | 11.35 |
| *g__Faecalibaculum* | 2.992 | 0.0004446 | 0.005717 | 0 | 0.01056 | 0.02795 | 0.005002 |
| *g__NK4A214_group* | 2.364 | 0 | 0.1613 | 0.0005558 | 0.04835 | 0.1493 | 0.3968 |
| *g__norank_f__norank_o__Clostridia_UCG-014* | 2.27 | 0.3691 | 0.3271 | 0 | 0.05669 | 0.2052 | 0.9199 |
| *g__UCG-005* | 2.199 | 0.01734 | 0.693 | 0 | 0.5108 | 0.4389 | 2.567 |
| *g__Marvinbryantia* | 2.112 | 0.05825 | 0.498 | 0 | 0.6664 | 1.157 | 1.472 |
| *g__Erysipelatoclostridium* | 1.685 | 1.295 | 0.05209 | 0.0005558 | 0.02223 | 0.03112 | 0.2462 |
| *g__Ruminococcus* | 1.538 | 0.06403 | 1.265 | 0.001667 | 0.8187 | 1.009 | 1.585 |

Table S4 (Continuous) The proportion of genera in each group exceeds 1%.

| Species name | NS-ord  -Mean(%) | ABX-ord  -Mean(%) | NS-fat  -Mean(%) | ABX-fat  -Mean(%) | FMT_Diab  -Mean(%) | FMT_Non  -Mean(%) | FMT_Con  -Mean(%) |
| --- | --- | --- | --- | --- | --- | --- | --- |
| *g__Phascolarctobacterium* | 1.511 | 0 | 0.0921 | 0 | 0.2262 | 0.1093 | 0.2245 |
| *g__norank_f__Eubacterium_coprostanoligenes_group* | 1.494 | 0.006225 | 0.2623 | 0 | 0.1795 | 0.5812 | 1.816 |
| *g__Parabacteroides* | 1.251 | 0 | 0.3557 | 0 | 0.2451 | 0.2966 | 0.07281 |
| *g__Lachnospiraceae_NK4A136_group* | 1.103 | 0.0008893 | 0.002541 | 0 | 0.003891 | 0.01016 | 0.004446 |
| *g__Erysipelotrichaceae_UCG-003* | 1.022 | 0.173 | 0.2249 | 0 | 0.1106 | 0.1505 | 0.09782 |
| *g__Klebsiella* | 0.009337 | 40.14 | 0.1086 | 92.32 | 0.4435 | 1.487 | 0.03724 |
| *g__Ruminococcus_gnavus_group* | 0.007559 | 5.538 | 0.6034 | 0 | 1.561 | 0.1524 | 0.01501 |
| *g__Enterococcus* | 0.01823 | 4.863 | 0.1004 | 0.00667 | 0.02612 | 0.05209 | 0.02112 |
| *g__Escherichia-Shigella* | 0.05869 | 3.251 | 1.662 | 2.442 | 0.8087 | 0.9496 | 0.01667 |
| *g__Parasutterella* | 0.06092 | 2.546 | 0.01461 | 0.02112 | 0.02112 | 0.01906 | 0.01945 |
| *g__unclassified_o__Lactobacillales* | 0.003557 | 1.709 | 0.01906 | 0.001667 | 0.0005558 | 0.002541 | 0.000556 |
| *g__Lachnoclostridium* | 0.3121 | 0.4313 | 4.815 | 0.001112 | 4.48 | 4.895 | 8.377 |
| *g__Ruminococcus_torques_group* | 0.4731 | 0.3246 | 3.71 | 0 | 9.978 | 5.809 | 3.473 |
| *g__norank_f__Lachnospiraceae* | 0.3304 | 0.003112 | 2.117 | 0 | 1.458 | 1.882 | 3.634 |

Table S4 (Continuous) The proportion of genera in each group exceeds 1%.

| Species name | NS-ord  -Mean(%) | ABX-ord  -Mean(%) | NS-fat  -Mean(%) | ABX-fat  -Mean(%) | FMT_Diab  -Mean(%) | FMT_Non  -Mean(%) | FMT_Con  -Mean(%) |
| --- | --- | --- | --- | --- | --- | --- | --- |
| *g__Anaerostipes* | 0.03913 | 0.07203 | 1.529 | 0 | 1.376 | 1.093 | 0.1062 |
| *g__Bacteroides* | 0.9835 | 0.03424 | 1.435 | 0 | 2.29 | 1.262 | 0.5308 |
| *g__Subdoligranulum* | 0.001779 | 0.6012 | 1.037 | 0.0005558 | 0.1184 | 1.778 | 0.05892 |
| *g__norank_f__Butyricicoccaceae* | 0.1805 | 0.0578 | 0.7578 | 0.0005558 | 2.003 | 0.7337 | 0.8398 |
| *g__Coriobacteriaceae_UCG-002* | 0.4602 | 0.0004446 | 0.7133 | 0 | 1.448 | 1.208 | 1.595 |
| *g__Fusicatenibacter* | 0.06581 | 0.8639 | 0.1277 | 0 | 1.157 | 0.5259 | 0 |
| *g__Adlercreutzia* | 0.5954 | 0 | 0.4364 | 0 | 0.8587 | 1.392 | 0.4502 |
| *g__Collinsella* | 0.06136 | 0 | 0.3487 | 0.0005558 | 0.1478 | 1.121 | 0.3357 |
| *g__Fournierella* | 0.1152 | 0.0004446 | 0.5666 | 0 | 0.6136 | 0.7584 | 1.576 |
