# Supplementary figures and images for: The relationship between gut microbiota and susceptibility to type 2 diabetes mellitus in rats
Source: Chin Med. 2023 May 5;18:49. doi: 10.1186/s13020-023-00717-9 (PMC10161507; doi:10.1186/s13020-023-00717-9)

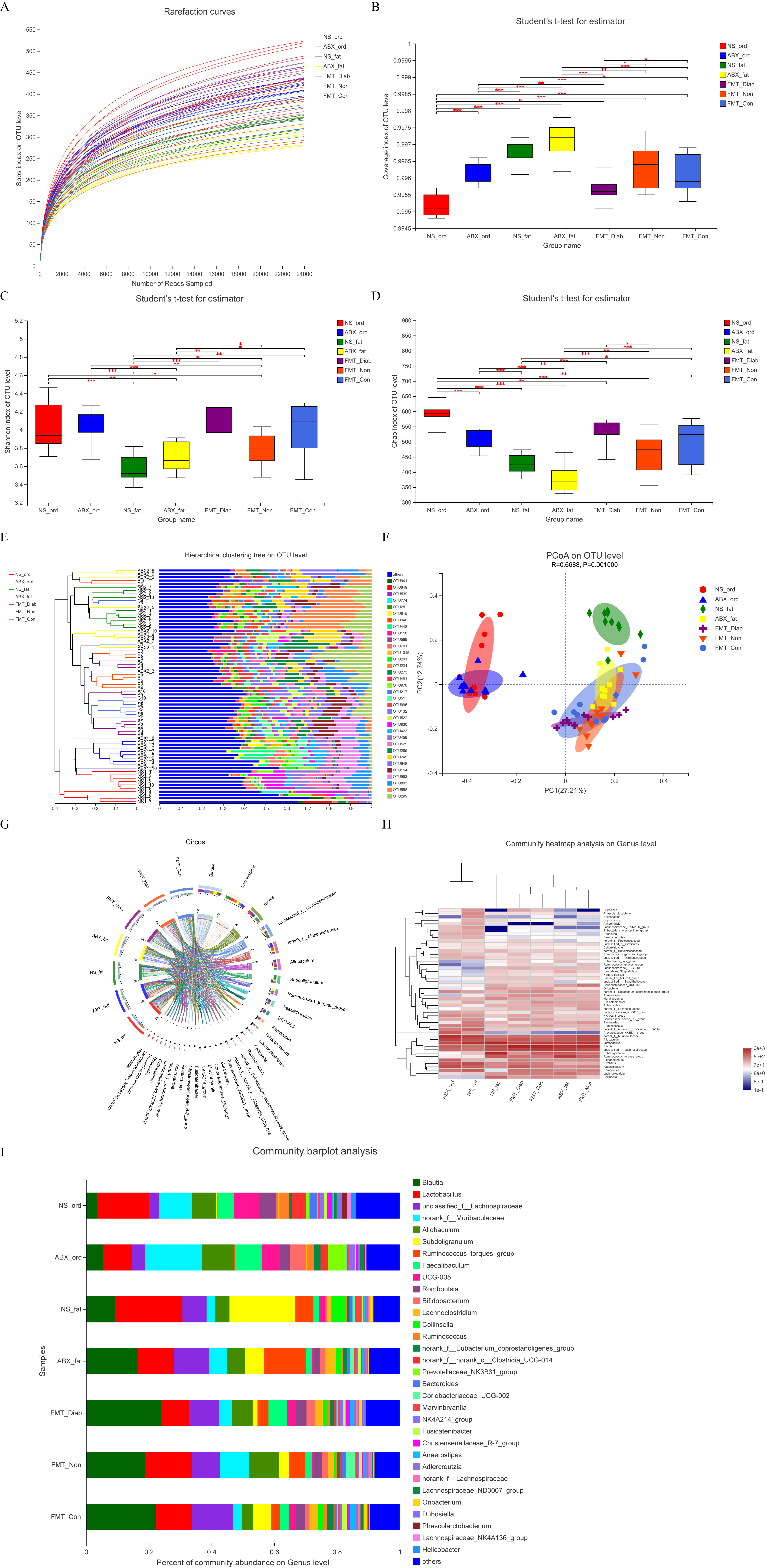

Supplement: Supplementary file 3 — Additional file 3: Figure S2. Diversity of fecal microbiota in the NS-ord, ABX-ord, NS-fat, ABX-fat, FMT-Diab, FMT-Non and FMT-Con groups. A Rarefaction curve. B–D Bacteria that were different between the NS and ABX groups in the B Coverage, C Shannon index and D Chao index. Differences were assessed by the Wilcoxon rank-sum test. *P < 0.05, **P < 0.01. E Hierarchical clustering tree at the operational taxonomic unit (OTU) level. F Principal co-ordinate analysis (PCoA) at the OTU level. G Circos sample–species relation map. H Community heatmap analysis at the genus level. I Community barplot analysis. [file 13020_2023_717_MOESM2_ESM.tif]

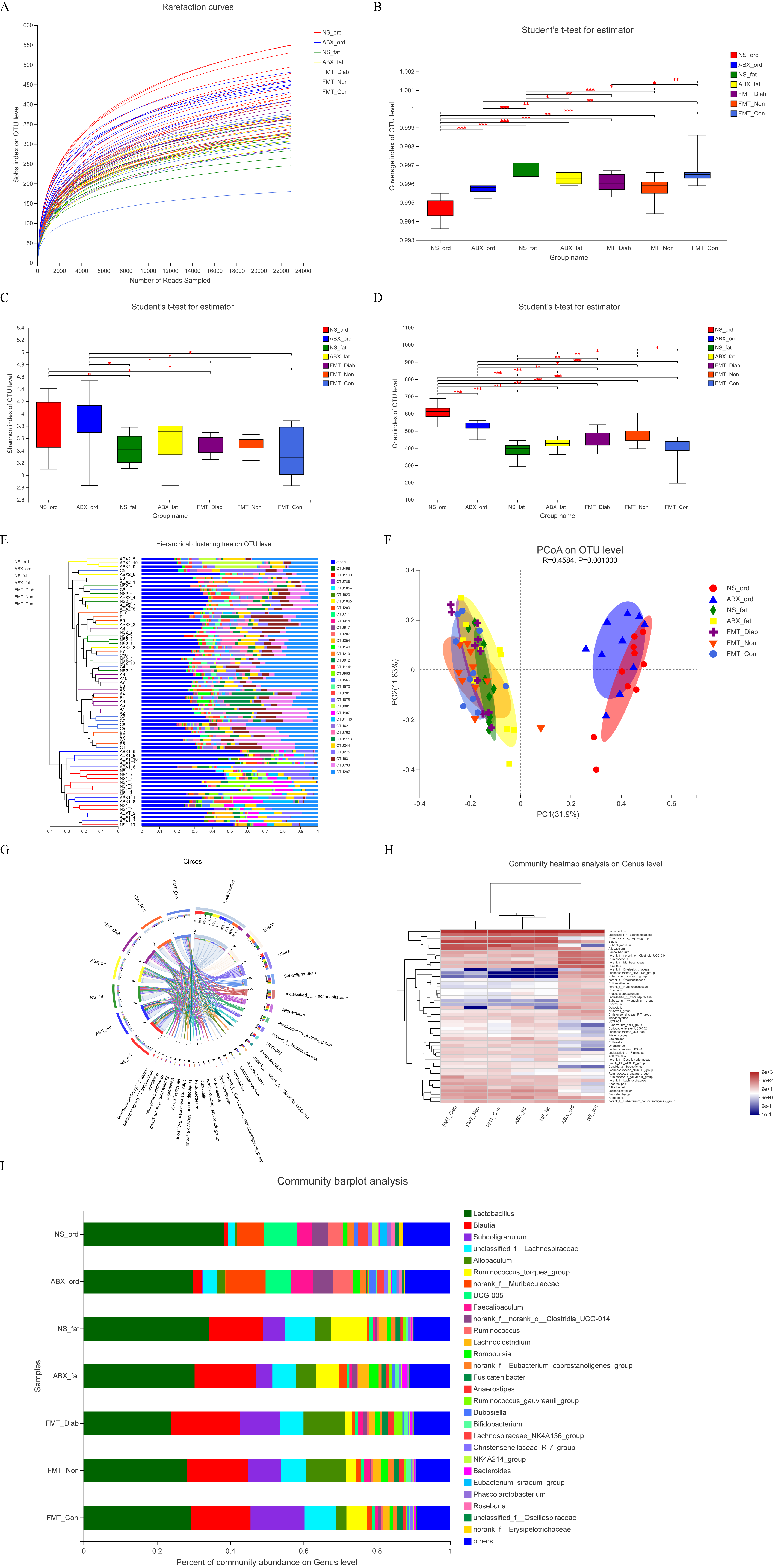

Supplement: Supplementary file 4 — Additional file 4: Figure S3. Diversity of fecal microbiota in the NS-ord, ABX-ord, NS-fat, ABX-fat, FMT-Diab, FMT-Non and FMT-Con groups. A Rarefaction curve. B–D Bacteria that were different among the groups in the B Coverage, C Shannon index and D Chao index. Differences were assessed by the Wilcoxon rank-sum test. *P < 0.05, **P < 0.01. E Hierarchical clustering tree at the operational taxonomic unit (OTU) level. F Principal co-ordinate analysis (PCoA) at the OTU level. G Circos sample–species relation map. H Community heatmap analysis at the genus level. I Community barplot analysis. [file 13020_2023_717_MOESM3_ESM.tif]

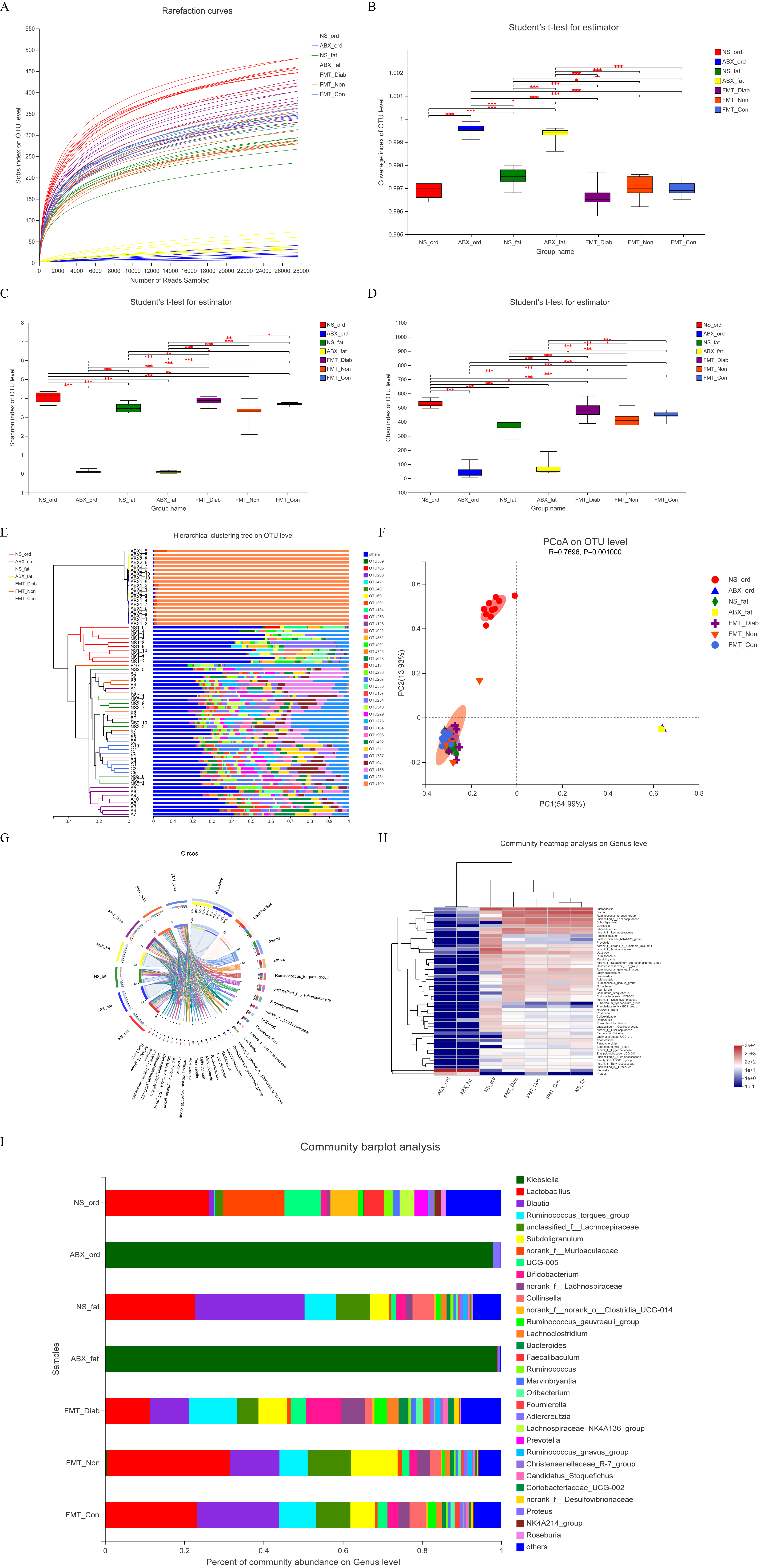

Supplement: Supplementary file 5 — Additional file 5: Figure S4. Diversity of fecal microbiota in the NS-ord, ABX-ord, NS-fat, ABX-fat, FMT-Diab, FMT-Non and FMT-Con groups. A Rarefaction curve. B–D Bacteria that were different among the groups in the B Coverage, C Shannon index and D Chao index. Differences were assessed by the Wilcoxon rank-sum test. *P < 0.05, **P < 0.01. E Hierarchical clustering tree at the operational taxonomic unit (OTU) level. F Principal co-ordinate analysis (PCoA) at the OTU level. G Circos sample–species relation map. H Community heatmap analysis at the genus level. I Community barplot analysis. [file 13020_2023_717_MOESM4_ESM.tif]
